# Antineoplastic effects of CPPTL via the ROS/JNK pathway in acute myeloid leukemia

**DOI:** 10.18632/oncotarget.17166

**Published:** 2017-04-17

**Authors:** Hui-Er Gao, Yue Sun, Ya-Hui Ding, Jing Long, Xiao-Lei Liu, Ming Yang, Qing Ji, Ying-Hui Li, Yue Chen, Quan Zhang, Ying-Dai Gao

**Affiliations:** ^1^ State Key Laboratory of Experimental Hematology, Institute of Hematology and Blood Diseases Hospital, Chinese Academy of Medical Sciences and Peking Union Medical College, Tianjin 300020, P. R. China; ^2^ State Key Laboratory of Medicinal Chemical Biology, College of Pharmacy, Nankai University, Tianjin 300353, P. R. China

**Keywords:** CPPTL, acute myeloid leukemia, ROS, JNK pathway

## Abstract

Drug resistance and human leukocyte antigen (HLA) matching limit conventional treatment of acute myeloid leukemia (AML). Although several small molecule drugs are clinically used, single drug administration is not sufficient to cure AML, which has a high molecular diversity. Metabolic homeostasis plays a key role in determining cellular fate. Appropriate levels of reactive oxygen species (ROS) maintain the redox system balance, and excessive amounts of ROS cause oxidative damage, thus providing a strategy to eliminate cancer cells. CPPTL is a novel analogue of parthenolide that exhibited significant cytotoxicity to AML cells *in vitro* and induced apoptosis in a dose-dependent manner. Additionally, CPPTL's prodrug DMA-CPPTL decreased the burden of AML engraftment and prolonged survival in a mouse model administered human primary AML cells *in vivo*. CPPTL induced apoptosis of AML cells by stimulating ROS production, and accumulation of ROS then activated the JNK pathway, thereby promoting mitochondrial damage. These results demonstrated that CPPTL effectively eradicated AML cells *in vitro* and *in vivo* and suggested that CPPTL may be a novel candidate for auxiliary AML therapy.

## INTRODUCTION

Acute myeloid leukemia (AML) is the most common hematological malignancy in adults [1]. Chemotherapy and hematopoietic stem cell (HSC) transplantation are conventional treatments for AML [2, 3]. However, approximately 30%-40% of AML patients cannot receive HSC transplantation, because of human leukocyte antigen (HLA) matching limitation. Moreover, the sequelae of autologous or allogeneic HSC transplantation can affect patient quality of life [4]. In addition, some patients are resistant to chemotherapeutics [5, 6], and high-dose chemotherapy drugs can cause severe side effects. Therefore, screening selective small molecule drugs for AML treatment is necessary and urgent.

Reactive oxygen species (ROS), which are small, highly reactive molecules, play key roles in the regulation of normal physiological processes and maintenance of the redox balance [7, 8]. Excessive generation of ROS causes cellular damage and apoptosis [9], which has been proposed as an approach to eliminate cancer cells. Stimulation of ROS generation has anti-tumor effects against cancers including lung cancer [10], gastric cancer [11], bladder cancer [12], cervical cancer [13], esophageal cancer [14]. Indeed, several novel small molecules that induce apoptosis through ROS-dependent pathways have been investigated in leukemic cells. Claudia P. Miller has reported that the proteasome inhibitor NPI-0052 induces T-cell acute lymphoblastic leukemia (T-ALL) and chronic myelogenous leukemia (CML) apoptotic cell death *in vitro* and *in vivo* by increasing the ROS levels [15]. Michele Milella has found that perturbation of AML cell redox status may play a role in the observed pro-apoptotic synergism between MEK inhibitors and retinoid [16]. Therefore, stimulating excessive ROS generation may be an adjuvant therapy for leukemia.

Apoptosis results from caspase activation and two well-studied evolutionarily conserved pathways: the cell surface death receptor pathway [17, 18] and the mitochondria-initiated pathway [19]. In the mitochondria-initiated pathway, the BCL-2 family, comprising antiapoptotic proteins, such as BCL-2, BCL-X_L_, and MCL-1, and proapoptotic proteins, such as BAX and BAK, have key roles in regulating mitochondrial membrane permeabilization. The ratio of these two types of proteins, such as BCL-2/BAX, controls the threshold of susceptibility to apoptosis [20]. If the balance of BCL-2/BAX is disrupted, and the ratio decreases, mitochondrial membrane potential (ΔΨm) decreases and induces release of cytochrome c. Cytochrome c and Apaf-1 recruit pro-caspase-9, thus facilitating formation of the apoptosome complex. Activated caspase-9 cleaves and then activates pro-caspase-3, which triggers apoptosis [20]. Poly (ADP-ribose) polymerase (PARP) is one of the major substrate of caspase-3 *in vivo*, and cleaved PARP facilitates cellular disassembly and induces apoptosis [21, 22].

Extending these previous findings, we herein report that the small molecule CPPTL exhibits cytotoxicity *in vitro* and decreases latency for AML development *in vivo*. Our study revealed that CPPTL induces apoptosis of AML cells by rapidly stimulating ROS generation, thereby rapidly triggering downstream caspase activation.

## RESULTS

### CPPTL inhibited leukemic cell proliferation and induced apoptosis

**Figure 1:**
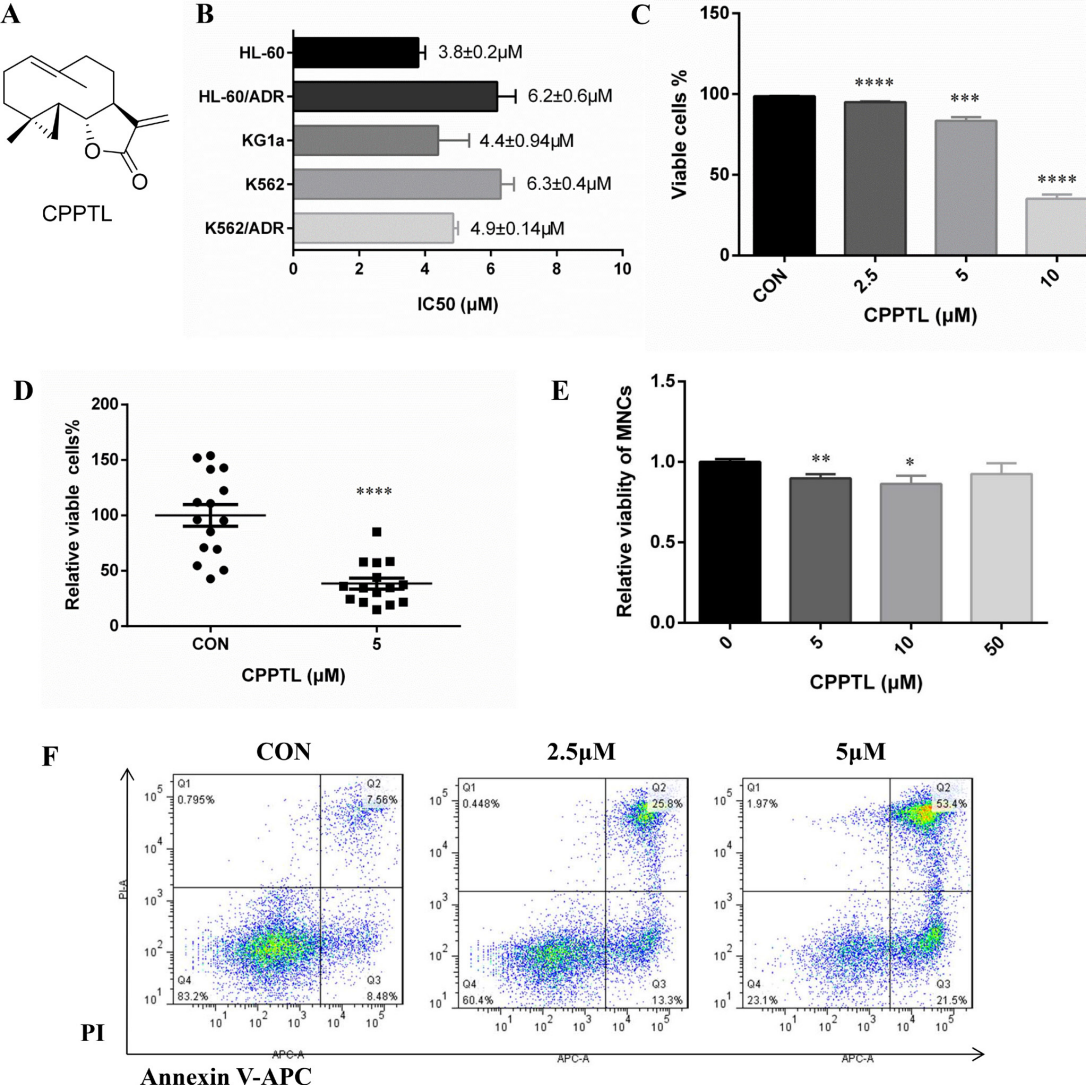
CPPTL inhibited leukemic cell proliferation and induced apoptosis (**A**) The chemical structure of CPPTL. (**B**) Cytotoxicity of cell lines exposed to CPPTL, as determined by MTT assays. We used MTT assays to determine the concentration that inhibited approximately 50% cell proliferation. All IC_50_ values are the average of three independent experiments. (**C**) Percentage of viable KG1a cells and (**D**) percentage of viable primary AML cells, assessed after treatment with CPPTL for 24 h. CON represents the vehicle control. (**E**) Percentage of apoptosis was assessed in MNCs isolated from human umbilical cord blood and treated with CPPTL for 24 h. (**F**) Representative flow cytometry images of primary AML samples treated with CPPTL. All data represent the mean ± SD. *p < 0.05, **p < 0.01, ***p < 0.005, ****p < 0.001.

CPPTL, whose chemical structure is shown in Figure 1A, is a cyclopropyl analogue of parthenolide [23]. To determine whether CPPTL had anti-leukemic effects, we determined its effects on five typical leukemia cell lines: HL-60 (APL cell line), HL-60/ADR (ADR-resistant APL cell line), K562 (CML cell line), K562/ADR (ADR-resistant CML cell line) and KG1a (AML cell line). CPPTL exhibited significant cytotoxicity to leukemic cells (Figure 1B). For sensitive cell lines, CPPTL was more specific to acute leukemia cells than chronic leukemia cells. Because HL-60 represents only acute promyelocytic leukemia (APL), we therefore selected KG1a to perform subsequent phenotypic and mechanistic studies. In particular, CPPTL induced apoptosis of KG1a and primary AML cells in a dose-dependent manner (Figure 1C and 1F). Moreover, we collected fifteen primary AML samples with different subtypes of AML to test the effects of CPPTL (Table 1). CPPTL, compared with the vehicle control, eliminated more than 70% of AML cells at 5 μM (Figure 1D). In contrast, CPPTL did not induce significant cytotoxic effects in normal mononuclear cells (MNCs) isolated from human umbilical cord blood *in vitro* (Figure 1E).

**Table 1:** Viability of primary AML samples treated with compound CPPTL

AML sample	subtype	Vehicle Control (%)			5 μM (%)		
		viable	early apoptosis	late apoptosis	viable	early apoptosis	late apoptosis
177643	AML-M3	23.4 ± 1.99	46.03 ± 1.93	29.9 ± 1.75	18.86 ± 0.37	50.46 ± 2.22	30.03 ± 1.7
217030	AML-M2a	60.57 ± 1.77	19.47 ± 1.03	15.97 ± 0.58	20.5 ± 0.44	26.43 ± 1.65	49.8 ± 1.65
225561	AML-M2a	27.65 ± 3.46	8.59 ± 0.33	62.8 ± 3.68	10.45 ± 0.66	10.67 ± 0.5	77.53 ± 1.07
231111	AML-M5	78.17 ± 0.47	9.3 ± 0.76	11.9 ± 0.35	19 ± 3.01	13 ± 2	66.17 ± 4.38
241596	AML-M5	38.8 ± 6.07	20.6 ± 1.06	39.43 ± 4.91	8.17 ± 1.2	24 ± 0.61	66.63 ± 1.35
241801	AML-M5	67 ± 2.29	9.68 ± 0.26	20.5 ± 1.91	11.77 ± 3.74	49.27 ± 1.4	37.83 ± 3.59
249515	AML-M2a	46.63 ± 3.72	38.8 ± 4.25	14.03 ± 1.16	13.4 ± 1.85	53.1 ± 3.39	32.83 ± 1.72
253390	AML-M5b	61.13 ± 5.71	20.8 ± 4.7	17.3 ± 1.37	31.67 ± 0.92	39.2 ± 1.71	28.57 ± 0.99
259800	AML-M5b	52.07 ± 1.19	23 ± 0.75	23.9 ± 1.44	31.93 ± 2.26	23.23 ± 1.32	42.63 ± 1.74
270896	AML-M4	84.23 ± 0.47	7.16 ± 0.64	7.29 ± 0.26	46.5 ± 0.78	38.27 ± 1.16	12 ± 0.72
288509	AML-M3a	29.83 ± 0.7	34.53 ± 1.13	34.8 ± 1.8	11.9 ± 0.52	58.27 ± 1.37	29.07 ± 0.81
315680	AML-M2a	52.57 ± 0.4	20.2 ± 0.92	25.3 ± 0.5	31.3 ± 0.4	29.17 ± 2.16	35.67 ± 1.46
315890	AML-M5	37.97 ± 1.25	12.63 ± 1.7	46.5 ± 2.05	24.13 ± 3.13	9.28 ± 2.19	59.57 ± 6.11
316582	AML-M1	77.43 ± 0.78	12.03 ± 0.57	9.01 ± 0.35	16.63 ± 0.78	38.2 ± 2.35	41.53 ± 0.83
326318	AML-M5	83.1 ± 0.17	8.39 ± 0.49	7.45 ± 0.29	19.73 ± 2.99	22.87 ± 1.65	54.97 ± 1.43

All samples were replicated three times.

### CPPTL decreased engraftment of primary AML samples and prolonged survival in a mouse model

**Figure 2:**
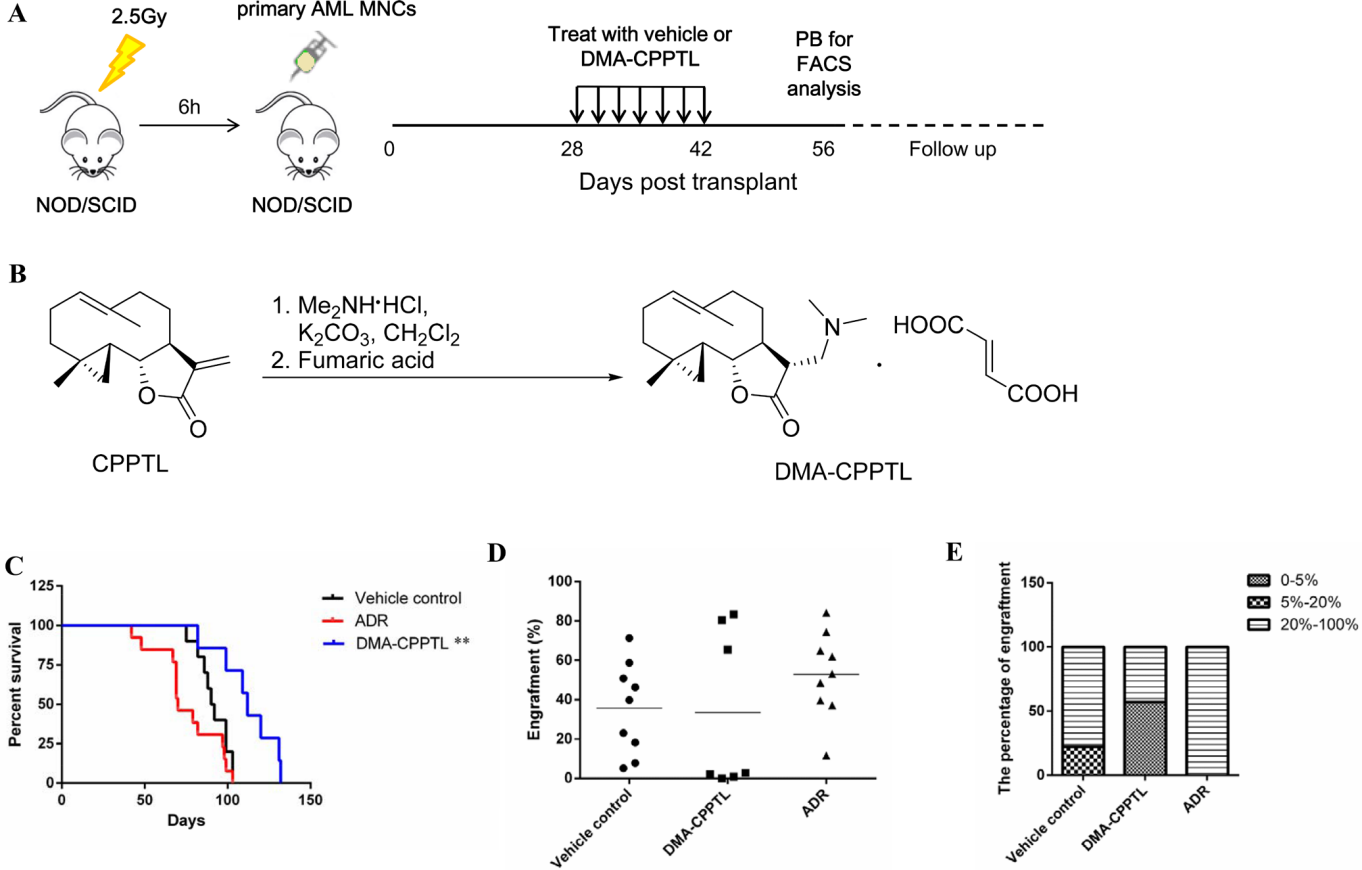
CPPTL eliminated AML cells *in vivo* and prolonged survival in a mouse model (**A**) Schematic of the xenotransplantation experiment. NOD/SCID mice received 2.5 Gy irradiation before transplantation of 1 × 10^7^ primary human AML MNCs via the tail vein. AML mice were randomly distributed to groups receiving 100 mg/kg of DMA-CPPTL via i.g. administration or vehicle every 48 h for 7 treatments. The positive control group received 2 mg/kg of ADR, a traditional chemotherapy drug, via i.v. injection every 72 h for 4 treatments. After 8 weeks, we detected CD45^+^ cells in peripheral blood to assess the success of AML engraftment. The survival curve shows only mice transplanted with AML successfully. (**B**) Synthesis of DMA-CPPTL. (**C**) Survival curves of 2.5 Gy-irradiated NOD/SCID mice transplanted with primary AML MNCs receiving treatment with DMA-CPPTL (*n* = 7, shown in blue), ADR (*n* = 9, shown in red) and vehicle control (*n* = 9, shown in black). (**D**) FACS analysis of the percentage of CD45^+^ cell engraftment in bone marrow after natural death of AML mice. (**E**) Percentage of mice exhibiting varying degrees of leukemic cell engraftment.

CPPTL exhibited significant anti-leukemic effects *in vitro*, which prompted us to evaluate the effects of CPPTL *in vivo* by using a NOD/SCID xenotransplantation mouse model. However, CPPTL showed weak solubility in water. Therefore, CPPTL was converted to its prodrug, DMA-CPPTL (Figure 2B). The protocol for the *in vivo* experiment is shown in Figure 2A. We transplanted 30 NOD/SCID mice with primary human AML MNCs and randomly divided into three groups immediately. As shown in Figure 2C, DMA-CPPTL, compared with the vehicle control and the positive control adriamycin (ADR), significantly prolonged the survival of mice. Flow cytometry was used to analyze the percentage of CD45^+^ cells in bone marrow after AML mice died naturally; the results indicated the engraftment level of AML. Compared with the vehicle control, the conventional chemotherapy drug ADR did not substantially prevent invasion of leukemic cells. However, the DMA-CPPTL treatment group exhibited lower engraftment of leukemic cells (Figure 2D). Half of the mice treated with DMA-CPPTL had lower levels of engraftment (<5%), but almost all ADR-treated mice exhibited high levels of engraftment (> 40%) (Figure 2E). These results suggested that CPPTL effectively eliminated leukemic cells *in vivo* and improved the survival of NOD/SCID mice transplanted with primary AML cells.

### CPPTL induced apoptosis by stimulating ROS generation

**Figure 3:**
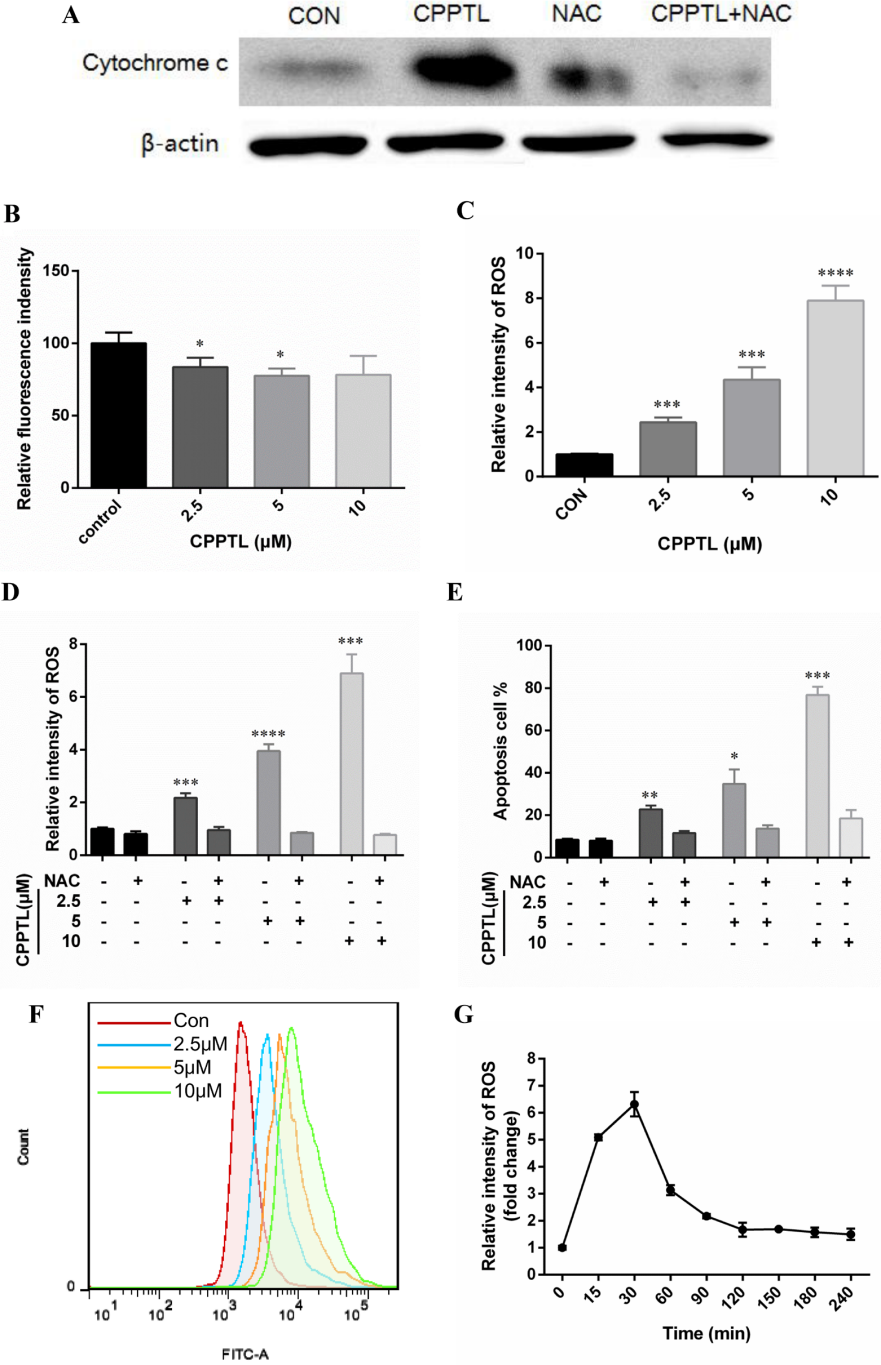
CPPTL induced apoptosis by stimulating ROS generation (**A**) Western blot analysis of KG1a cells treated with or without the ROS inhibitor NAC, or treated with NAC alone for 24 h. All lanes contained aliquots of protein extract samples. (**B**) ΔΨm levels were measured on the basis of TMRE fluorescence in KG1a cells treated with CPPTL for 24 h. (**C**) ROS levels were measured on the basis of DCF-DA fluorescence in KG1a cells treated with CPPTL for 1 h. (**D**) Representative flow cytometry images of ROS detection. (**E**) ROS levels in KG1a cells after co-treatment with CPPTL and NAC for 1 h. (**F**) Percentage of viable KG1a cells, assessed after co-treatment with CPPTL and NAC for 24 h. (**G**) ROS levels in KG1a cells treated with 5 μM CPPTL at different time points. All data represent the mean ± SD. *p < 0.05, **p < 0.01, ***p < 0.005, ****p < 0.001.

Apoptosis is involved in an increase in mitochondrial membrane permeability and release of cytochrome c, thus decreasing ΔΨm. A fluorescent dye, tetramethyl rhodamine ethyl-ester (TMRE), was used to examine ΔΨm with flow cytometry to quantify apoptosis. TMRE binds to the mitochondrial membrane only when ΔΨm is high; otherwise, it is released from the mitochondrial membrane. Cytochrome c was released (Figure 3A), and ΔΨm was decreased (Figure 3B) after CPPTL treatment, thus indicating that CPPTL induced mitochondria-initiated apoptosis. We detected ROS levels of KG1a cells after CPPTL treatment for 24 hours to determine whether CPPTL-induced apoptosis is facilitated by intracellular ROS accumulation. CPPTL stimulated ROS generation in a dose-dependent manner (Figure 3C and 3F), and N-acetylcysteine (NAC), a free radical scavenger, prevented CPPTL-induced ROS accumulation after co-treatment with CPPTL and NAC (Figure 3D). Moreover, NAC effectively inhibited apoptosis of KG1a cells induced by treatment with different concentrations of CPPTL (Figure 3E). Through monitoring the dynamic ROS levels, we found that in contrast to other ROS activators, CPPTL stimulated ROS production rapidly within 15 minutes. ROS levels peaked in 30 minutes, and subsequently, ROS levels decreased and were maintained at a certain level (Figure 3G). In summary, these data revealed that CPPTL stimulated production of ROS, thus inducing apoptosis.

### JNK activation contributed to CPPTL-induced apoptosis of AML cells

**Figure 4:**
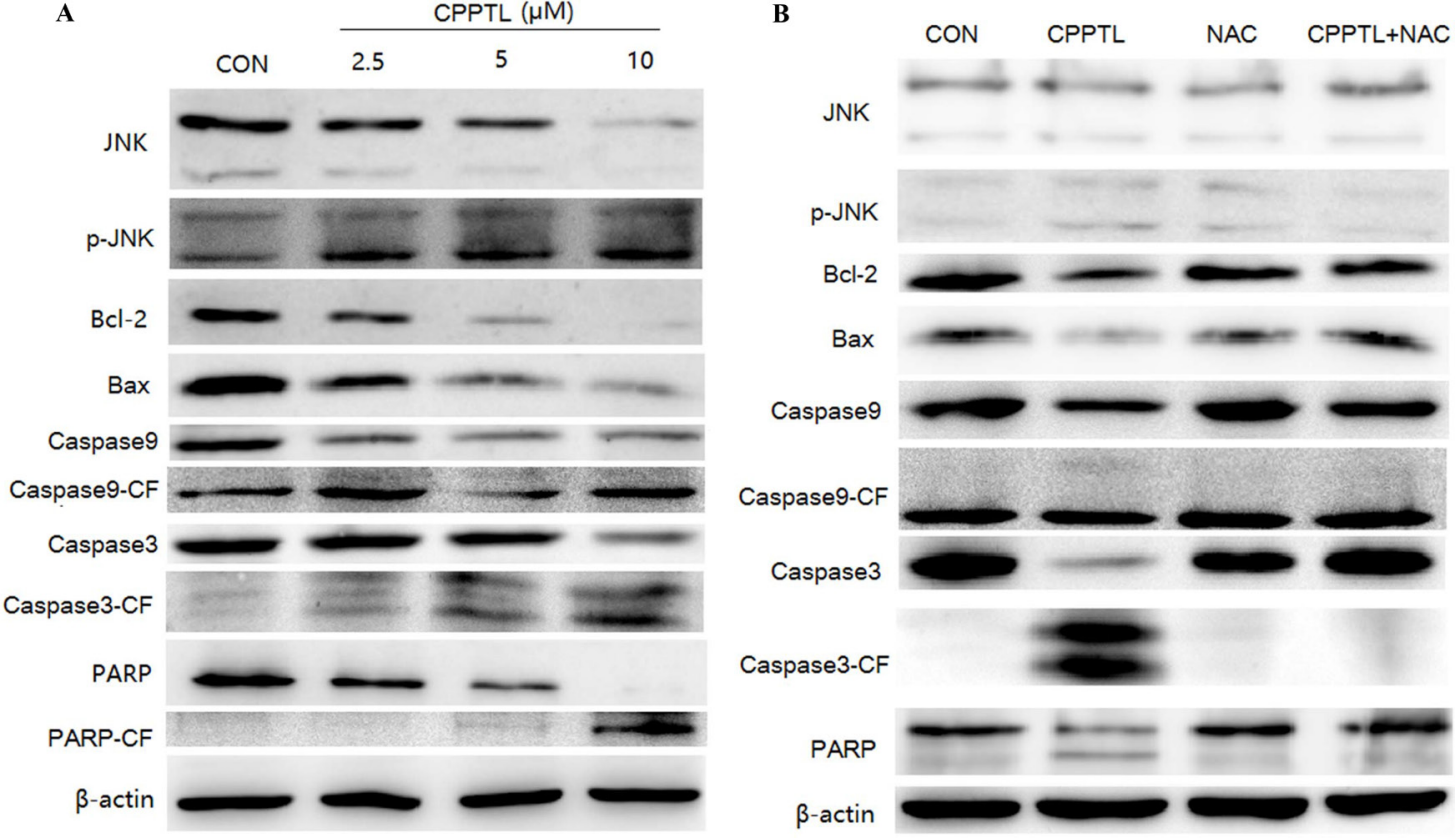
JNK activation contributed to CPPTL-induced apoptosis of AML cells Western blot analysis of KG1a cells (**A**) treated with different concentrations of CPPTL for 24 h, (**B**) treated with or without the ROS inhibitor NAC, or treated with NAC alone for 24 h. All lanes contained aliquots of protein extract samples.

Our study demonstrated that CPPTL promotes ROS production, thereby inducing apoptosis. Several factors are known to activate mitochondria-initiated apoptosis. However, the protein that is activated to trigger this series of reaction is not known. As shown in Figure 4A, CPPTL promoted phosphorylation of JNK and p38 and decreased expression of the anti-apoptotic protein Bcl-2 in a dose-dependent manner. Although the pro-apoptotic protein Bax also decreased, there was no effect on apoptosis of KG1a cells treated with CPPTL. Notably, cleavage of caspase-9, caspase-3 and PARP, key proteins associated with apoptotic activation, increased in a dose-dependent manner.

To validate the effects of ROS in this pathway, we used NAC to block ROS and then analyzed the protein expression. As shown in Figure 4B, NAC substantially blocked the CPPTL-induced phosphorylation of JNK and p38, the release of cytochrome c, the decrease in Bcl-2, and the cleavage of caspase-9, caspase-3 and PARP, but NAC had no effect on any of these events.

**Figure 5:**
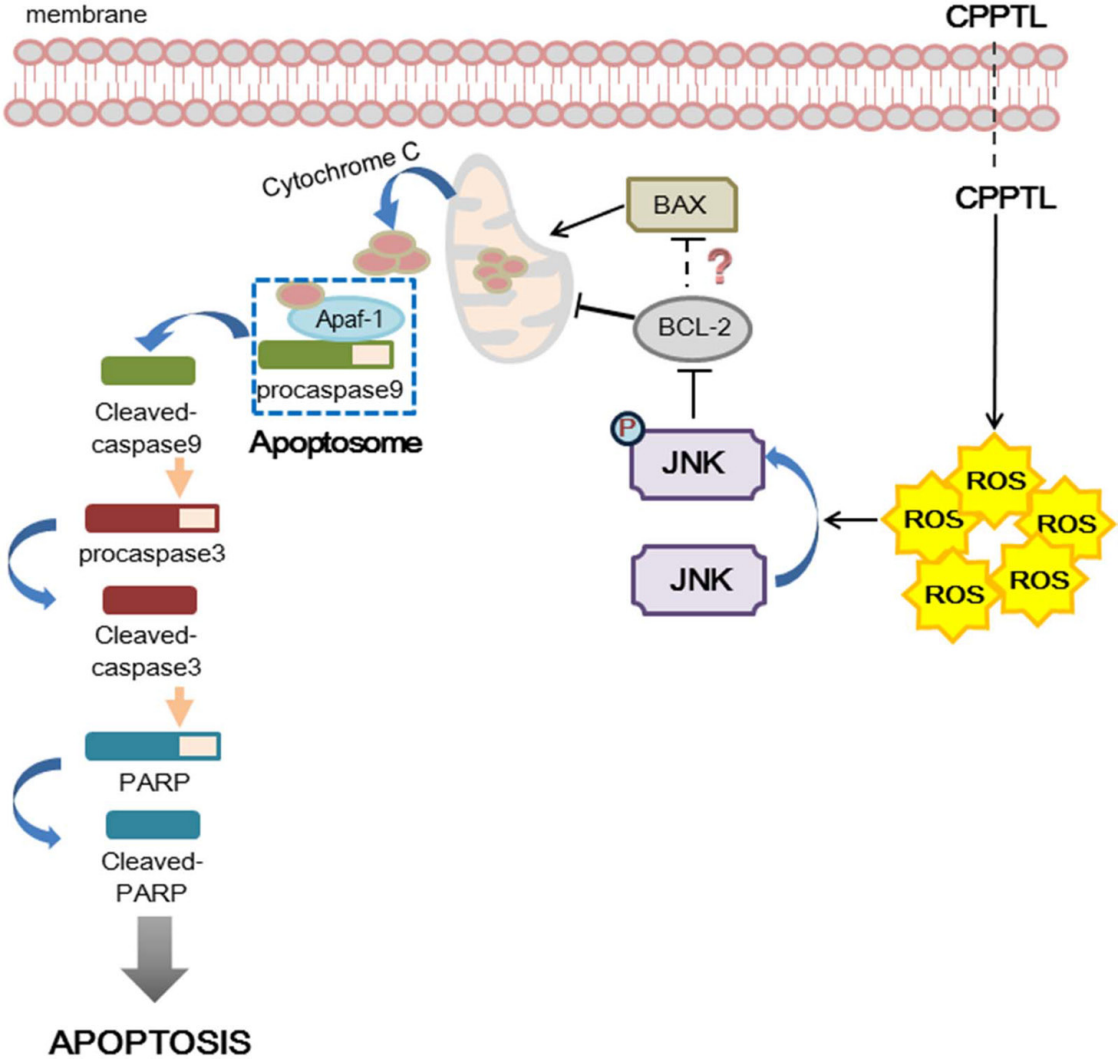
Hypothesized model of hierarchical signaling events leading to apoptosis of AML cells after treatment with CPPTL

As shown in Figure 5, we hypothesized that CPPTL induces apoptosis by inducing ROS generation, which subsequently leads to activation of JNK. Phosphorylated JNK may inhibit the anti-apoptotic effect of Bcl-2 and then promote release of cytochrome c from mitochondria. Free cytochrome c may bind to Apaf-1 and consequently recruit pro-caspase-9, thus allowing formation of the apoptosome. The apoptosome then cleaves caspase-9, activates pro-caspase-3 and PARP, and ultimately induces apoptosis.

## DISCUSSION

AML is the most common acute leukemia in adults, accounting for ~80 percent of cases in this group [24]. Given the complexity of AML classification, the most common treatments in clinical use are still chemotherapy and HSC transplantation. Nevertheless, researchers are still exploring the molecular mechanisms underlying the origin of AML to find more effective drug targets. In recent years, FLT3-ITD [25], STAT [26, 27] and IDH1/IDH2 [28] have been identified as effective therapeutic targets, and several inhibitors of these proteins are currently undergoing clinical trials. In addition, monoclonal antibodies [29] and CART therapy [30] remain research hotspots. Although novel targeted therapies hold promise for effective anti-leukemia treatment with low toxicity from off-target effects, single drug administration is insufficient to cure AML, which has a high molecular diversity. Therefore, combinations of different treatment methods and therapeutic targets are necessary.

In both normal cells and cancer cells, homeostasis of cellular metabolism determines the cellular fate. Indeed, disrupting this balance may be a potential therapeutic strategy to eradicate cancer cells. Increasing evidence indicates that ROS stress is different between normal cells and cancer cells. ROS are essential for biological functions and participate in signaling cell growth and differentiation, regulating the activity of enzymes, mediating inflammation by stimulating cytokine production, and eliminating pathogens [31, 32]. The effect of ROS on cancer cells is double-edged. A certain amount of ROS can promote the proliferation of cancer cells, but excessive amounts of ROS cause oxidative damage to kill the cancer cells [33]. Thus, manipulating ROS levels is a strategy to selectively eliminate cancer cells without causing toxicity to normal cells [34]. Therefore, our group has studied the relationship between ROS and leukemia and has screened novel agents targeting leukemia. On the basis of our previous studies, we evaluated a novel small molecule compound, CPPTL, and its anti-leukemia effect.

CPPTL exhibited significant anti-leukemic effects *in vitro* and *in vivo*. For common AML cell lines, CPPTL inhibited proliferation and induced apoptosis in a dose-dependent manner. Moreover, CPPTL was more specific to AML than CML. NOD/SICD mouse model assays demonstrated that DMA-CPPTL, a water soluble prodrug of CPPTL, decreased the capacity of primary AML sample xenotransplantation *in vivo*. DMA-CPPTL, compared with the vehicle control and positive control, prolonged the survival time significantly, and the percentage of CD45^+^ cell engraftment was lower. These results suggested that CPPTL may be a promising anti-leukemia drug candidate. In addition, we found that CPPTL induced apoptosis by stimulating ROS generation, thereby triggering JNK pathway activation. ROS levels were sustainably maintained at a certain level; nevertheless ROS increased rapidly after CPPTL treatment, within 15 min. Excessive ROS promotes phosphorylation of JNK and activates mitochondria-initiated apoptosis [35]. JNK has multiple targets, including Bcl-2 family members, which bind to the mitochondrial membrane [36]. In fact, phosphorylated JNK should promote Bax by inhibiting Bcl-2. However, in our western blot results, the Bax expression unexpectedly decreased. We hypothesized that although Bax expression decreased, Bcl-2 was decreased even more. Therefore the ratio of Bcl-2/Bax also decreased and triggered ΔΨm alteration, thus inducing release of cytochrome c. After cytochrome c was free from the mitochondria, apoptosis was induced.

Moreover, CPPTL exhibited a strong ability to kill drug-resistant CML cells compared with sensitive CML cells, thus suggesting that CPPTL might overcome drug-resistant chronic myelogenous leukemia (CML). Indeed, it has been reported that ROS has a key role in inducing apoptosis or death in drug-resistant cancer cells. Muhammad Altaf *et al*. have synthesized new bipyridine gold(III) dithiocarbamate-containing complexes that have potent anticancer capacity against cisplatin-resistant cancer cells by increasing ROS levels [37]. In addition to solid tumors, the novel agent RRx-001 overcomes drug resistance in multiple myeloma cells via release of ROS to activate caspase [38]. More importantly, in 2007, Jennifer S. Carew *et al*. have reported that the combination of chloroquine and SAHA augments anticancer activity by targeting autophagy, thus overcoming Bcr-Abl-mediated drug resistance, and the generation of ROS plays an important role during this process [39].

ROS play a decisive role in eradicating AML stem cells [40]. AML stem cells tend to have a lower metabolic activity and thus lower ROS production than AML cells overall. Lagadinou Eleni D has demonstrated that the majority of AML stem cells are enriched in ROS-low cell populations, and Bcl-2 is overexpressed in this group [41]. Several compounds target leukemia stem cells through ROS production [41–45]. Integration of ROS with other key LSC signaling interference strategies had powerful toxic effects on AML stem cells [40]. Our group has focused on the effects of small molecules on AML and AML stem cells through ROS production [46, 47]. In addition, compared with other AML cell lines, KG1a cells exhibit a CD34^+^CD38^−^ LSC-like phenotype. Collectively, CPPTL may have the potential to target LSC selectively. Thus, our future research will focus on the effects of CPPTL on drug-resistant CML and AML stem cells.

In conclusion, we report that CPPTL can eliminate AML cells *in vitro* and *in vivo*. CPPTL activates the JNK pathway and consequently induces mitochondria-initiated apoptosis by stimulating ROS production. In conclusion, our results suggested that modulation of ROS levels may be used as an auxiliary method of AML treatment. However, the detailed mechanisms underlying CPPTL function have not been elucidated. Therefore, more in-depth research and optimization should be carried out in future studies.

## MATERIALS AND METHODS

### Cell lines, primary samples and cell culture

We obtained KG1a, HL-60, K562, HL-60/ADR, K562/ADR cell lines from the State Key Laboratory of Experimental Hematology (Tianjin, China) and cultured them in RPMI 1640 supplemented with 10% FBS (HyClone, USA) at 37°C in 5% CO_2_. Primary human AML samples were collected from the Institute of Hematology & Blood Diseases Hospital. Mononuclear cells were isolated from each sample using Ficoll-Plaque density gradient separation and were cryopreserved in 90% FBS/10% dimethyl sulfoxide (DMSO). Primary human AML samples were retrieved and cultured in serum-free Iscove's modified Dulbecco's medium (IMDM).

### Synthesis of CPPTL and DMA-CPPTL

CPPTL was synthesized according to our previously reported procedure [23]. The compound was dissolved in DMSO and stored at −80°C at a concentration of 20 mM. The compound was diluted with relevant medium for cellular experiments.

A mixture of CPPTL (590 mg, 2.4 mmol), Me_2_NH.HCl (2.94 g, 36 mmol), CH_2_Cl_2_ (120 mL), and K_2_CO_3_ (9.95 g) was heated to reflux for 5 h. The reaction mixture was filtered. The filtrate was concentrated under reduced pressure to yield a crude solid, which was purified on silica gel column, thus yielding a white solid (645 mg). The resulting solid was dissolved in methanol, and then, fumaric acid (257 mg) was added. The mixture was stirred 10 min at room temperature and then concentrated under reduced pressure to give DMA-CPPTL (902 mg, 92%). ^1^H NMR (400 MHz, MeOD) δ 6.70 (s, 2H), 5.20–5.23 (m, 1H), 4.03 (t, *J* = 8.2 Hz, 1H), 3.53 (dd, *J* = 13.3, 10.0 Hz, 1H), 3.37 – 3.31 (m, 1H), 3.07 (dt, *J* = 12.9, 5.0 Hz, 1H), 2.94 (s, 6H), 2.54 – 2.39 (m, 1H), 2.31 – 2.10 (m, 3H), 1.98–2.02 (m, 2H), 1.96 – 1.85 (m, 2H), 1.72 (s, 3H), 1.04 (s, 3H), 0.97 – 0.80 (m, 2H), 0.56 (dd, *J* = 9.3, 4.7 Hz, 1H), 0.38 (t, *J* = 5.2 Hz, 1H); ^13^C NMR (100 MHz, MeOD) δ 177.9, 171.0, 136.2, 134.3, 128.6, 87.9, 57.3, 52.1, 45.5, 44.3, 41.7, 39.7, 32.5, 32.0, 26.2, 21.5, 18.7, 18.5, 16.8.

### MTT cell proliferation assay

Briefly, leukemia cells were seeded in 96-well plates at a density of 5×10^3^cells/ well. Cells were treated with CPPTL at a concentration gradient for 72 h. After treatment, MTT was added at a final concentration of 0.5 mg/ml and allowed to react with cells for 4 h. The formazan precipitate was dissolved in DMSO, and the absorbance was read at 570 nm.

### Apoptosis assays

Apoptosis of KG-1a cells and primary human AML samples was detected with an Annexin V-FITC/PI Apoptosis Detection Kit (SUNGENE BIOTECH, China). KG-1a cells were cultured in 24-well plates at a density of 1 × 10^5^cells/well for 24 h and treated with CPPTL alone or together with 5 mM NAC. Primary human AML samples were cultured in 24-well plates treated with CPPTL for 24 h, at a density range from 2×10^5^ to 5×10^5^cells/well depending on the total cell numbers of different samples. After 24 h, cells were washed with cold phosphate-buffered saline (PBS), re-suspended in 1×binding buffer and incubated with Annexin V-FITC in the dark for 10 min. PI was added 5 min before detection. All samples were analyzed with a BD LSRFortessa flow cytometer (BD Biosciences, USA).

### Mitochondrial membrane potential (ΔΨm)

Mitochondrial membrane potential changes were qualified with a TMRE-Mitochondrial Membrane Potential Assay Kit (Abcam, USA). After treatment of CPPTL for 24 h, TRME was added in KG1a culture medium and incubated for 30 min at 37°C, 5% CO_2_. Collected cells were re-suspended in PBS/0.2% BSA. All samples were analyzed with a BD LSRFortessa flow cytometer (BD Biosciences, USA) with the PE channel.

### ROS detection

ROS levels were detected with a Reactive Oxygen Species Assay Kit (Beyotime, China). Before CPPTL treatment, DCFH-DA was added in the culture medium at a final concentration of 10 μM. Then, 1 × 10^5^ KG1a cells were seeded in 24-well plate with medium containing DCFH-DA and treated with CPPTL. After treatment, the cells were collected and washed three times with phosphate-buffered saline (PBS). All samples were analyzed with a BD LSRFortessa flow cytometer (BD Biosciences, USA).

### Western blotting

KG1a cells (1 × 10^6^) were incubated with the indicated concentrations of CPPTL and washed with PBS, then lysed for 30 min on ice. The supernatant was centrifuged and collected to determine the total protein concentration by using BCA reagent (Thermo Fisher Scientific, America). Aliquots of protein extract samples (~20 μg) were separated on 12% sodium dodecyl sulfate (SDS) - polyacrylamide gels and then transferred to nitrocellulose membranes and blocked with 5% skim milk for 2 h at room temperature. Membranes were incubated with primary antibodies diluted 1:1000 in PBST at 4°C overnight and then washed three times with PBST before being incubated with the corresponding secondary antibodies for 2 h at room temperature (1:20,000 dilution with PBST). The immunoreactive bands were detected with Western Lightning Plus-ECL reagent (PerkinElmer, USA) according to the manufacturer's instruction. The following primary antibodies were used: anti-β-actin (Sigma-Aldrich, USA), anti-Bcl-2 (#4223), anti-Phospho-p38 (#4511), anti-Bax (#5023), anti-Caspase-9 (#9502), anti-PARP (#9532), and anti-Caspase-3 (#9665), all of which were from Cell Signaling Technology, USA.

### Statistical analysis

All statistical analyses were performed with GraphPad Prism 6 software. Data are expressed as the mean ± standard deviation (SD). Student's *t* test was used for determining the differences between two groups. *P* values < 0.05 were considered statistically significant.

## Abbreviations

ADR: Adriamycin
AML: Acute myeloid leukemia
APL: Acute promyelocytic leukemia
CML: Chronic myelogenous leukemia
HLA: Human leukocyte antigen
HSC: Hematopoietic stem cell
MNCs: Mononuclear cells
MTT: Methyl thiazolyl tetrazolium
NAC: N-acetylcysteine
PARP: Poly (ADR-ribose) polymerase
ROS: Reactive oxygen species
T-ALL: T-cell acute lymphoblastic leukemia
TMRE: Tetramethyl rhodamine ethyl-ester
